# Effects of exercise training on carotid intima-media thickness in patients with type 2 diabetes and coronary artery disease. Influence of carotid plaques

**DOI:** 10.1186/s12933-016-0336-2

**Published:** 2016-01-22

**Authors:** Rune Byrkjeland, Knut-Haakon Stensæth, Sigmund Anderssen, Ida U. Njerve, Harald Arnesen, Ingebjørg Seljeflot, Svein Solheim

**Affiliations:** Center for Clinical Heart Research, Department of Cardiology, Oslo University Hospital Ullevål, PO box 4956, Nydalen, 0424 Oslo, Norway; Center for Heart Failure Research, Oslo University Hospital, Oslo, Norway; Faculty of Medicine, University of Oslo, Oslo, Norway; Oslo University Hospital Ullevål, Oslo, Norway; Department of Sports Medicine, Norwegian School of Sport Sciences, Oslo, Norway

**Keywords:** Type 2 diabetes, Coronary artery disease, Exercise training, Carotid intima-media thickness, Atherosclerosis

## Abstract

**Background:**

Carotid intima-media thickness (cIMT) holds prognostic information for future cardiovascular disease and is associated with the extent of coronary atherosclerosis. We investigated the effect of exercise on cIMT progression in patients with both type 2 diabetes and coronary artery disease (CAD).

**Methods:**

Patients with type 2 diabetes and CAD (n = 137) were randomized to exercise training or standard follow-up. The 12 month exercise program contained 150 min weekly of combined aerobic and resistance training. High-resolution ultrasonography of the distal part of the common carotid artery (CCA) was performed to measure cIMT before and after the intervention. The CCA and the carotid bulb were scanned for the presence of atherosclerotic plaques. Differences in changes between the randomized groups were calculated by one-way ANCOVA.

**Results:**

In the total population no difference in changes of cIMT from baseline to 12 months was observed between the exercise group and controls [−0.016 mm (95 % CI −0.037 to 0.006) vs. −0.007 mm (95 % CI −0.029 to 0.015), p = 0.57]. However, there was a significant interaction between the effect of exercise training and the presence of carotid plaques (p = 0.013), and significant reduced cIMT was demonstrated in the exercise group compared with controls in patients without identified carotid plaques (n = 65) [−0.034 mm (95 % CI −0.060 to 0.008) vs. 0.013 mm (95 % CI −0.011 to 0.038), p = 0.010].

**Conclusion:**

One year of exercise training in patients with type 2 diabetes and CAD did not significantly change cIMT progression. However, in patients without identified carotid plaques, beneficial effect of exercise training on cIMT progression was demonstrated.

## Background

Carotid intima-media thickness (cIMT) is an established marker of cardiovascular risk. Several studies have shown associations between cIMT and the risk for future cardiovascular events in both healthy individuals, patients with type 2 diabetes and patients with known coronary artery disease (CAD) [1–3]. cIMT has also been used as a surrogate marker of generalised atherosclerosis and studies have shown associations between cIMT and the extent of atherosclerosis in the coronary arteries [4, 5]. Further, Hodis et al. showed that the progression of cIMT in patients with established CAD was predictive of coronary events and argued that cIMT changes in these patients reflected their underlying atherosclerotic progression [1].

Previous studies in patients with type 2 diabetes have shown reduced progression of cIMT after treatment of cardiovascular risk factors like hyperglycemia and hypertension, and changes in cIMT have been associated with changes in HbA1c [6]. Physical activity over long time may protect against atherosclerosis in healthy individual [7, 8], and in type 2 diabetes exercise and lifestyle intervention may improve cardiovascular risk factors and attenuate cIMT progression [9, 10].

In patients with known CAD, previous studies with lifestyle and exercise interventions have shown attenuated progression of coronary atherosclerosis [11, 12], although more recent exercise trials have indicated less anti-atherosclerotic effect of exercise in patients on statin treatment [13, 14].

Patients with type 2 diabetes have increased cIMT and their atherosclerotic disease is more accelerated and widespread compared to non-diabetic patients [6, 15, 16]. Dyslipidemia and alterations in reverse cholesterol transfer, partly on genetic basis, may contribute to this [17–20]. Not many studies have investigated effects of exercise on cIMT or other measures of atherosclerosis in patents with both type 2 diabetes and CAD, and whether exercise has beneficial effect on the progression of atherosclerosis beyond up-to-date medical treatment in these patients is not clear.

The aim of the present study was therefore to investigate the effect of exercise training on cIMT progression in patients with the combination of type 2 diabetes and CAD. We hypothesised that exercise training would reduce the progression of cIMT in these patients.

## Methods

### Study design and participants

This study is part of a randomized clinical trial investigating effects of exercise training on cardiovascular disease (CVD) risk factors and measures of atherosclerosis in patients with type 2 diabetes and CAD (ClinicalTrials.gov: NCT01232608). Patients with known type 2 diabetes and verified CAD by coronary angiography (n = 137) were included at the Department of Cardiology, Oslo University Hospital, Ullevål, Oslo, Norway between August 2010 and March 2012. The last follow-up was in March 2013. Exclusion criteria were presence of proliferative retinopathy, end stage renal disease, cancer, stroke or acute myocardial infarction within the last 3 months, unstable angina, uncompensated heart failure, serious arrhythmia, severe valvular disease, severe rheumatologic disease, chronic obstructive pulmonary disease stadium GOLD IV, thromboembolic disease, ongoing infections, severe musculoskeletal disorders and other disabilities limiting the ability for physical activity. All patients gave informed, written consent to participate in the study. The study was approved by The Regional Ethics Committee and was conducted according to the Declaration of Helsinki. At the time of inclusion the patients were randomized in a 1:1 ratio to either the exercise group or the control group. The control group continued with standard follow-up by their general practitioner. Study design and methods have also previously been reported [21].

### Exercise intervention

The study participants randomized to the exercise group underwent a 12 months combined aerobic and resistance training program planned and conducted in collaboration with the Norwegian School of Sports Sciences, Oslo, Norway. The exercise program consisted of group-based exercise sessions of 60 min duration twice a week under supervision of qualified instructors throughout the intervention period, and a third weekly home-based individual exercise session. The total exercise volume was 150 min per week, of which approximately two-thirds was aerobic and one-third resistance exercises. The exercise sessions were guided by Borg’s scale of rated perceived exertion (RPE) and included parts of high intensity interval training (RPE ≥ 15) [21].

### Anthropometric and laboratory measures

Body weight was measured with light clothing on the same weight before and after the intervention. Waist circumference was measured horizontally at the level of the iliac crest and at the end of a normal expiration. Arterial blood pressure (BP) was measured automatically in the brachial artery with patients in a supine position (Riester ri-champion^®^ N, Jungingen, Germany). Blood samples were drawn at inclusion and end of the study by standard venipuncture between 0800 and 1000 AM after overnight fast, without medication taken since the preceding evening and without exercise training the last 24 h. The final blood samples were taken within 1 week after the last exercise section. Total cholesterol, high-density lipoprotein (HDL) cholesterol, triglyserides were determined by conventional laboratory methods. Low-density lipoprotein (LDL) cholesterol was calculated by Friedewalds formula. HbA1c was measured by turbidimetric inhibition immunoassay (Roche, Basel, Switzerland) and CRP was determined by high sensitivity enzyme-linked immunosorbent assay (DRG Instruments, Marburg/Lahn, Germany).

### Common carotid artery ultrasonography

We implemented a standardized protocol and strict quality control procedures to achieve reliable ultrasonic measurements of the carotid artery IMT [22]. The subjects were studied in the morning under standardized conditions (quiet room, comfortable temperature) and in the fasting state. One experienced sonographer who was blinded to the participants’ group status and risk factor levels did all the examinations. High-resolution ultrasonography was performed with a Zonare z.one ultra (Zonare medical Systems, Mountain View, CA, USA) ultrasound scanner equipped with electrocardiogram (ECG) and a linear array 5–10 MHz transducer, using 8 MHz as centre frequency and utilizing the harmonic frequencies. The participants were examined in the supine position with the head turned slightly to both sides. After identifying the bulb, longitudinal images of the common carotid artery (CCA) were obtained by combined B-mode and colour Doppler. The scan was focused on the far wall segment 20 mm proximal of the carotid bulb, where all measurements were done. All images were acquired by using an anterior oblique angle (30° from midline), and/or if needed, a lateral angle (100° from midline) [23, 24]. Measurements were taken during end-diastole (R-wave of the ECG) [25]. The CCA and the carotid bulb region were also scanned for the presence of atherosclerotic plaques, defined as a distinct area of the vessel protruding >50 % of the adjacent parts of the intima-media layer [26]. cIMT was measured at locations without atherosclerotic plaques, although plaques could be present in adjacent areas. The plaques’ volume and content (calcified, non-calcified, or mixed plaque) were analysed at baseline. Calculation of plaque volumes was based on manually set measurements of length, width and height of the plaques. All scans were digitally stored on the internal hard disk for subsequent off-line analysis using Osirix MD (v. 2.5.1) (Pixmeo, Geneva, Switzerland). Three end-diastolic frames were selected, and the mean cIMT was calculated for each subject as the average of maximum 9 (3 × 3) consecutive measurements on each side. A minimum of two measurements on one side were needed to be included in the baseline analyses and at least two measurements from the same side at both baseline and 12 months to be included in the interval analyses. The intrasonographer variability has been shown earlier [22] [correlation coefficient for mean cIMT 0.99 (0.977–0.999)].

### Statistical analysis

The power calculations in the main study were based on an expected 10 % relative reduction of HbA1c in the exercise group while unchanged in the control group. Accounting for possible dropouts the calculations indicated a minimum of 68 patients in each arm of the study [21].

Demographic data are given as proportions, mean (±SD) or medians (25, 75 percentiles) with skewed data. Between group differences in baseline characteristics were calculated by independent-sample Students t-test, Chi square test or Mann–Whitney test as appropriate. Baseline associations were assessed by Pearson correlation or Spearman’s rank correlation as appropriate. Differences between the randomized groups in changes from baseline to 12 months were calculated by one-way ANCOVA. Skewed data distributions were log transformed before entering the model. Within group changes in cIMT was calculated by paired-samples Student’s t-test. Changes are presented as mean [95 % confidence interval (CI)]. Interactions between the exercise effect on cIMT and relevant baseline factors were investigated by two-way ANCOVA. Based on clinical judgement, the following factors were considered potential effect modifiers (continuous variables were dichotomized at median values): Gender, etnicity (caucasian), age (>64), body mass index (BMI) (>29 kg/m^2^), previous myocardial infarction, presence of carotid plaques, years of known diabetes (>9), presence of microvascular complications, baseline LDL (>2.0 mmol/L) and baseline HbA1c (>7.4 %). SPSS version 18.0 for Windows was used. p value < 0.05 was defined as statistically significant.

## Results

Flow chart of the study population, dropouts and exercise adherence have been reported in detail elsewhere [21]. In summary, 123 patients completed the study with eight dropouts in the exercise group and six in the control group. Baseline characteristics of the study subjects are shown in Table 1. No patients were in NYHA class > II (angina or dyspnea). Due to plaque burden and technical challenges baseline cIMT measurements were determined in 117 patients, whereas valid measurements of changes in cIMT (according to the protocol) were obtained in 104 patients. There were no significant differences between the randomized groups at baseline, and baseline characteristics did not differ between patients included in the cIMT analyses or not.

**Table 1:** Baseline characteristics of all included patients and patients completing the study in the randomized groups (n = 123)

Characteristics	All (n = 137)^a^	Exercise (n = 61)	Control (n = 62)	p value
Age (years)	63.1 ± 7.9	63.5 ± 8.0	63.2 ± 7.2	0.82
Gender (male/female), n	115/22	54/7	50/12	0.34
Ethnicity (Caucasian/non-Caucasian), n	114/23	53/8	52/10	0.83
Current smoker, n (%)	23 (17)	10 (16)	9 (15)	0.97
Previous smokers, n (%)	65 (47)	30 (50)	30 (50)	1.00
BMI (kg/m^2^)	29.2 ± 5.0	29.2 ± 4.0	29.0 ± 5.6	0.80
Waist circumference (cm)	106.1 ± 12.7	106.7 ± 11.2	105.3 ± 14.2	0.55
Years of diabetes^b^	9 (5, 15)	10 (5, 15)	9 (5, 13)	0.48
Microvascular complications^c^, n (%)	55 (40)	24 (39)	23 (37)	0.94
Hypertension, n (%)	100 (73)	45 (74)	46 (74)	1.00
Angina pectoris, n (%)	51 (37)	25 (41)	26 (42)	1.00
Previous myocardial infarction, n (%)	62 (45)	25 (41)	31 (50)	0.41
Years since myocardial infarction^b^	3 (1, 11)	3 (1, 11)	3 (1, 11)	0.92
Previous PCI, n (%)	92 (67)	37 (61)	46 (74)	0.16
Previous CABG, n (%)	38 (28)	17 (28)	19 (31)	0.89
Chronic heart failure, n (%)	11 (8)	2 (3)	5 (8)	0.45
NYHA class II, n (%)	30 (22)	9 (15)	18 (29)	0.090
HbA1c (%)^b^	7.4 (6.8, 8.3)	7.3 (6.8, 8.3)	7.3 (6.8, 7.9)	0.90
Creatinine (μmol/L)^b^	79 (68, 91)	78 (69, 91)	67 (81, 88)	0.92
Medication, n (%)				
Anti-platelet therapy	129 (94)	56 (92)	61 (98)	0.20
Statins	128 (93)	57 (93)	58 (94)	1.00
Beta-blockers	106 (77)	49 (80)	48 (77)	0.86
ACE-inhibitors/ARB	97 (70)	39 (64)	46 (74)	0.30
CCB	45 (33)	19 (31)	21 (34)	0.85
Metformin	101 (74)	47 (77)	46 (74)	0.87
Sulfonylurea	48 (35)	25 (41)	17 (27)	0.16
Gliptins	17 (12)	6 (10)	11 (18)	0.31
Insulin/insulin-analogues	26 (19)	10 (16)	12 (19)	0.85

Data are presented as proportions or mean ± SD if not otherwise stated

*BMI* body mass index, *PCI* percutanous coronary intervention, *CABG* coronary artery bypass grafting, *NYHA* New York Heart Association functional classification of dyspnea, *ACE* angiotensin converting enzyme, *ARB* angiotensin receptor II blocker, *CCB* calcium channel blockers

p value refers to between group differences at baseline in patients who completed the study

^a^No significant between group differences

^b^Median (25, 75 percentiles)

^c^Defined as known diabetes microvascular complications, albuminuria (including micro-) and/or positive monofilament test at baseline

### Carotid IMT and plaques at baseline

The overall mean cIMT at baseline was 0.866 mm ± 0.180 in males (age 63.3 ± 7.8), and 0.793 mm ± 0.102 in females (age 61.7 ± 8.8). Baseline cIMT was correlated to age in men (n = 115) (r = 0.343, p < 0.001), but did not reach statistical significance in women (n = 22) (r = 0.321, p = 0.17). There was a significant correlation between cIMT and LDL cholesterol in men (r = 0.222, p = 0.023), but not in women (p = 0.92).

Fifty-eight patients (50 %) were identified as having carotid plaques. Of these, 42 patients had a single dominating plaque with a median volume of 97 mm^3^ (63, 142). These single plaques were classified as calcified (n = 9), non-calcified (n = 9) or mixed (n = 24). There was no significant difference in the presence of plaques between the randomized groups (p = 0.18). Patients with plaques were older (65.5 years ± 6.7 vs. 61.5 years ± 7.8, p = 0.003), had a lower proportion of non-Caucasians (5.2 % vs. 23.1 %, p = 0.011), higher systolic BP (143 mmHg ± 16 vs. 136 mmHg ± 16, p = 0.012) and higher cIMT (0.905 mm ± 0.195 vs. 0.826 mm ± 0.139, p = 0.014) compared to patients without plaques.

### Changes in cIMT

We could not demonstrate any significant difference between the exercise group and control group in changes of cIMT from baseline to 12 months in the overall population [−0.016 mm (−0.037 to 0.006) vs. −0.007 mm (−0.029 to 0.015), p = 0.57] (Table 2). The non-significant reduction of cIMT in the exercise group (p = 0.19), correlated with change in systolic BP (r = 0.279, p = 0.041) and tended to correlate with change in HbA1c (r = 0.254, p = 0.064), but not with change in LDL cholesterol (p = 0.55).

**Table 2:** Carotid intima-media thickness, blood pressure and serum lipids at baseline and after 12 months

	All (n = 137)^a^	Exercise (n = 61)		Control (n = 62)		p value
	Baseline	Baseline	12 months	Baseline	12 months	
cIMT (mm)	0.855 ± 0.172	0.876 ± 0.187	0.858 ± 0.175	0.853 ± 0.141	0.849 ± 0.131	0.57
SBP (mmHg)	139 ± 17	139 ± 17	140 ± 17	139 ± 16	139 ± 16	0.75
DBP (mmHg)	79 ± 9	78 ± 9	79 ± 9	80 ± 9	80 ± 10	0.51
TC (mmol/L)^b^	3.9 (3.4, 4.5)	3.8 (3.4, 4.3)	3.9 (3.3, 4.6)	4.0 (3.3, 4.7)	3.7 (3.2, 4.5)*	0.014
LDL (mmol/L)^b^	2.0 (1.6, 2.6)	1.9 (1.6, 2.5)	2.0 (1.7, 2.7)*	2.1 (1.6, 2.7)	1.9 (1.5, 2.7)*	0.020
HDL (mmol/L)^b^	1.12 (0.94, 1.40)	1.12 (0.96, 1.32)	1.11 (0.93, 1.41)	1.13 (0.93, 1.40)	1.09 (0.94, 1.34)	0.080
TG (mmol/L)^b^	1.40 (1.07, 1.88)	1.43 (1.09, 1.91)	1.53 (1.10, 1.99)	1.38 (0.98, 1.89)	1.38 (0.87, 2.01)	0.20
CRP (mg/L)^b^	2.41 (1.15, 4.99)	2.04 (0.95, 4.40)	1.99 (1.00, 4.21)	2.40 (1.19, 4.98)	1.77 (0.96, 3.86)	0.91

Data are presented as mean ± SD if not otherwise stated

*cIMT* carotid intima-media thickness, *SBP/DBP* systolic/diastolic blood pressure, *TC* total cholesterol, *LDL* low density lipoprotein cholesterol, *HDL* high density lipoprotein cholesterol, *TG* triglycerides. *CRP* c-reactive protein

p value refers to difference between the groups in changes from baseline to 12 months (one-way ANCOVA). * p < 0.05, within-group changes from baseline to 12 months

^a^No significant between group differences

^b^Median (25, 75 percentiles)

**Fig. 1:**
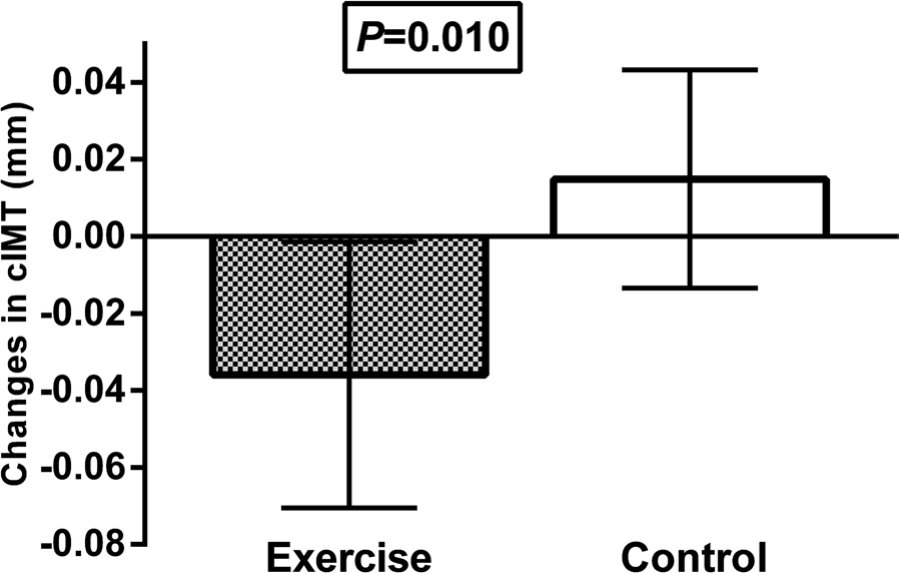
Absolute change in carotid intima-media thickness (cIMT) (mm) in patients without carotid plaques (n = 65) according to the randomized groups, illustrated as mean (95 % confidence interval). p value refers to the difference in changes from baseline to 12 months between the groups (one-way ANCOVA)

The identification of carotid plaques interacted with the effect of exercise on cIMT progression (Table 3). In the strata of patients without plaques, cIMT decreased significantly in the exercise group compared to controls [−0.034 mm (−0.060 to −0.008) vs. 0.013 mm (−0.011 to 0.038), p = 0.010] (Fig. 1), still significant after adjusting for CVD risk factors at baseline (smoking, LDL cholesterol, systolic blood pressure, HbA1c, gender and age) (p = 0.003). There was no difference between patients with and without carotid plaques in exercise adherence during the intervention (p = 0.68).

**Table 3:** Interaction with exercise effect on carotid intima-media thickness

	p value
Gender	1.00
Etnicity (Caucasian)	0.99
Age (>64)^a^	0.97
BMI (>29 kg/m^2^)^a^	0.20
Previous myocardial infarction	0.70
Presence of carotid plaques^b^	0.013
Years of known diabetes (>9)^a^	0.20
Microvascular complications^c^	0.71
LDL (>2.0 mmol/L)^a^	0.21
HbA1c (>7.4 %)^a^	0.28

*BMI* body mass index, *LDL* low density lipoprotein cholesterol

p value for interaction calculated by two-way ANCOVA

^a^Median value

^b^Detected by scanning of the common carotid artery and the carotid bulb region during ultrasonography

^c^Defined as known diabetes microvascular complications, albuminuria (including micro-) and/or positive monofilament test at baseline

### Changes in cardiovascular risk factors

We have previously reported no overall significant changes in the exercise group compared to controls in HbA1c [−0.22 % (−0.49 to 0.04) vs. −0.01 % (−0.25 to 0.24), p = 0.24], weight [−0.9 kg (−1.8 to 0.1) vs. 0.3 kg (−0.6 to 1.2), p = 0.087] or waist circumference (0.9 cm (−0.4 to 2.3) vs. 2.3 cm (1.0 to 3.6), p = 0.153) [21]. We could also not demonstrate any significant differences between the randomized groups in changes of BP, HDL cholesterol or CRP, whereas LDL cholesterol increased in the exercise group compared to controls (Table 2). When analysed according to the presence of carotid plaques, there were no differences in changes of the risk factors, except for LDL cholesterol levels which increased in the exercise group compared to controls in patients with plaques [0.20 mmol/L (0.00 to 0.41) vs −0.13 mmol/L (−0.37 to 0.12), p = 0.044], but not in patients without plaques (p = 0.40).

## Discussion

The main finding in this study was that 1 year of exercise training did not significantly change cIMT progression in patients with type 2 diabetes and CAD. However, the identification of carotid plaques interacted with the exercise effect and in patients without plaques cIMT decreased significantly in the exercise group compared to controls. These results indicate minor benefits of exercise training on the progression of atherosclerosis beyond up-to-date medical treatment in patients with type 2 diabetes and CAD. Nevertheless, local severity of atherosclerosis, assessed by the presence of carotid plaques in this study, may influence the effects of exercise, and attenuated progression seems to occur in vascular areas without advanced atherosclerotic disease.

### No overall change in cIMT progression

Exercise training has numerous beneficial effects in patients with either type 2 diabetes or CAD, and exercise plays an important role in the management of type 2 diabetes and in cardiac rehabilitation [9, 27, 28]. However, there is limited data on the effect of exercise in high-risk, complicated patients with the combination of type 2 diabetes and CAD.

Our main result indicates limited effects of exercise on cIMT progression in these patients beyond up-to-date medical treatment. This is in accordance with previous studies showing that the use of statins may influence the effect of exercise on the progression of cIMT/atherosclerosis. In statin treated patients with either CAD or hypertension, studies have not demonstrated reduced cIMT progression after exercise training [14, 29]. A recent study with multifactorial lifestyle intervention in patients with both type 2 diabetes and CAD also showed no effect on progression of atherosclerosis evaluated by intravascular ultrasound, although endothelial function did improve [30].

On the other hand, studies of healthy individuals and type 2 diabetic patients without statin treatment have demonstrated reduced progression of cIMT after exercise or lifestyle intervention [10, 13]. Lifestyle interventions in patients with CAD before the statin era also showed reduced progression of atherosclerosis [11, 12]. These results indicate that lipid-lowering treatment with statins may attenuate the additional benefits of exercise on cIMT/atherosclerosis progression, and this may have contributed to the overall neutral result in our study.

Other pharmacological treatments may also influence cIMT progression and thus the result of the present trial. Studies in patients with type 2 diabetes have shown that aspirin, enalapril, calcium channel blockers and metformin in combination with glibenclamide, may attenuate cIMT progression, independent of their impact on glycemic control and BP [6, 31].

We observed only small changes in CVD risk factors in the present study. Systolic BP and HbA1c did not decrease significantly, and as previous studies in patients with type 2 diabetes have shown associations between improvements of BP and HbA1c and reduction of cIMT progression [6, 31], this may have contributed to the minor change in cIMT. We confirmed these associations in the present study. The levels of CRP did also not change during the study, indicating that the exercise intervention did not have a significant anti-inflammatory effect in this population.

LDL cholesterol increased significantly in the exercise group compared to controls. This result stands in contrast to a minor reduction that could be expected after exercise in patients with type 2 diabetes only [9]. However, in patients with CAD or multiple cardiovascular risk factors metaanalyses of exercise interventions have not demonstrated beneficial change in LDL cholesterol [32, 33]. The patients in the present study all had CAD and type 2 diabetes, and most of them several other CVD risk factors in addition. Taken together, this may indicate that exercise has less beneficial, or even unbeneficial, effect on LDL cholesterol in patients with more complicated vascular disease. Interestingly, our subgroup analyses showed increased LDL cholesterol in patients with identified carotid plaques, but not in patients without plaques. Nevertheless, we observed no correlation between the changes in LDL cholesterol and cIMT, implying that the increase in LDL cholesterol did not influence cIMT progression during the study period. Others have also suggested that changes in LDL cholesterol levels are more strongly related to changes in plaque characteristics than to changes in absolute cIMT [6, 34].

The exercise modality in the present study may have contributed to the overall neutral result. Kadoglou et al. showed that only aerobic training ameliorated cIMT in patients with type 2 diabetes, and not resistance training or combined aerobic and resistance training [35].

Cesar et al. also discussed the limitations of exercise only to reduce established atherosclerosis. In an animal model they demonstrated that exercise is beneficial to reduce atherosclerosis only when combined with dietary intervention [36].

### Reduced cIMT in patients without carotid plaques

The identification of carotid plaques in the present study indicates severe atherosclerotic disease in adjacent vascular areas from where cIMT was measured. The presence of such plaques interacted with the exercise effect on cIMT, and we observed reduced cIMT in the exercise group compared to controls in patients without plaques. These results indicate that exercise may reduce cIMT/atherosclerosis development in vascular areas with no or early stages of atherosclerotic disease, whereas the anti-atherosclerotic effects of exercise is attenuated in areas with advanced atherosclerotic lesions. This may be discussed along with previous studies demonstrating paradoxical vasoconstriction during exercise in coronary arteries with significant atherosclerotic disease, whereas vasodilation occurred as expected in coronary arteries without signs of atherosclerosis [37, 38]. These results imply that the presence of atherosclerotic plaques interferes with endothelial function and disturbs the normal vasodilatory effect of exercise, possibly through reduced production and release of nitric oxid (NO). Knowing that NO also induces important anti-atherosclerotic responses, a possible explanation of our subgroup results is that the carotid vascular segments with plaques experience weaker NO-mediated effects of exercise compared to areas without plaques, where these beneficial responses to a greater extent are preserved.

To summarise, our results indicate that 12 months of combined aerobic and resistance exercise training without dietary intervention does not reduce progression of atherosclerosis in up-to-date medical treated patients with both type 2 diabetes and CAD. However, the local severity of atherosclerosis may influence the exercise effect and an attenuation of further atherosclerotic development may occur in less affected vascular areas. Thus, our results do not undermine the overall importance of physical activity and exercise, and patients with type 2 diabetes and CAD should be encouraged to regular exercise. Nevertheless, the results implicate that the anti-atherosclerotic effects of exercise are the most pronounced with milder or earlier stages of atherosclerotic disease.

## Limitations

The follow-up time in the present study was 12 months and this is relatively short time when studying the development and progression of atherosclerotic disease. Physical exercise may also be considered a “weak” intervention that require extra-long follow-up. Thus, the duration of our study may have been too short for an actual difference between the groups to occur.

Another limitation of the study was that atherosclerosis was only evaluated at one specific vascular site. As the progression of atherosclerosis may vary between different vascular areas, this limit the interpretation of the results at an individual level.

The power calculations in the main study were based on an expected change in HbA1c and this may have made it underpowered for the investigation of cIMT progression.

## Conclusion

One year of exercise training in patients with both type 2 diabetes and CAD did not significantly change cIMT progression. Up-to-date medication and minor changes in CVD risk factors during the intervention period may have contributed to this result. However, the identification of carotid plaques interacted with the effect of exercise on cIMT and a significant reduction in cIMT progression was demonstrated in patients without plaques. This indicates that the severity of local carotid atherosclerosis may influence the effect of exercise on cIMT progression and that beneficial change could be expected with less severe atherosclerotic disease.

## Abbreviations

cIMT: carotid intima-media thickness
CAD: coronary artery disease
CCA: common carotid artery
BP: arterial blood pressure
CRP: c-reactive protein
LDL: low-density lipoprotein cholesterol
HDL: high-density lipoprotein cholesterol
BMI: body mass index
CVD: cardiovascular disease
NO: nitric oxid
